# Epidemiology and Predictors of all-cause 30-Day readmission in patients with sickle cell crisis

**DOI:** 10.1038/s41598-020-58934-3

**Published:** 2020-02-07

**Authors:** Vivek Kumar, Neha Chaudhary, Maureen M. Achebe

**Affiliations:** 1 Department of Internal Medicine and Medical Oncology, Brigham and Women’s Hospital and Dana Farber Cancer Institute, Boston, USA; 2 Department of Pediatrics and Neonatology, Beth Israel Deaconess Medical Center, Boston, USA; 3 Division of Hematology, Brigham and Women’s Hospital, and Dana Farber Cancer Institute, Boston, USA

**Keywords:** Epidemiology, Anaemia, Sickle cell disease

## Abstract

The 30-day readmission rate after hospitalization for a sickle cell crisis (SCC) is extremely high. Accurate information on readmission diagnoses, total readmission costs and factors associated with readmission is required to effectively plan resource allocation and to plan interventions to reduce readmission rates. The present study aimed to examine readmission diagnoses and factors associated with all-cause 30-day readmission after hospitalization for SCC. We analyzed 2016 nationwide readmission database (NRD) to identify patterns of 30-day readmission by patient demographic characteristics and time after hospitalization for SCC. We estimated the percentage and most common readmission diagnoses for 30-day and 7-day readmissions after discharge. We studied the relationship between risk factors and readmission and the impact of readmission on patient outcomes and resulting financial burden on health care in dollars. In 2016, of 67,887 discharges after index hospitalizations, 18099 (26.9%) were readmitted within 30-days. Of all readmissions, 5166 (7.6%) were readmitted within 7 days. The spectrum of readmission diagnoses was largely similar in both 30-day and 7-day readmission with more than 80% patients in both time periods readmitted with diagnoses related to SCC. The mean length of stay for readmitted patients was significantly longer than the index hospitalization (5.3 days (5.1–5.5) vs 4.9 days (CI 4.8–5.1, p < 0.01). Also, the mean cost of hospitalization in readmitted patients $8485 was significantly higher than the index hospitalization $8064 p < 0.01. In 2016, readmission among patients with SCC incurred an additional 95,445 hospitalization days resulting a total charge of $609 million and a total cost of $152 million in the US. On Multivariate analysis, age group 18–30 years, discharge against medical advice, higher Charlson comorbidity index, low socioeconomic status and admission at high volume centers were associated with a higher likelihood of 30-day readmission. Among patients hospitalized for SCC, 30-day readmissions were frequent throughout the month post hospitalization and resulted in an enormous financial burden on the United States healthcare system.

## Introduction

Hospital readmissions impose a huge financial burden on healthcare systems and increase patients’ morbidity and mortality. The Medicare payment advisory commission estimates suggest that Medicare alone could have saved $12 billion per year by reducing hospital readmissions^1–3^. The Centers for Medicare & Medicaid services (CMS) consider all-cause 30-day readmission rates a quality metric of care by hospitals. Acute pain episodes or sickle cell crisis (SCC) (less favored term now) are the most common cause for seeking care among individuals with sickle cell disease (SCD). With the use of penicillin prophylaxis, hydroxyurea and better care of hospitalized patients, the survival of individuals with SCD has improved considerably. Based on estimates from 2010, 100,000 individuals with SCD live in the United States^4,5^.

Understanding the diagnoses and timing of readmissions is critical to formulate interventions to reduce readmissions whereas information on the added economic burden is required for efficient allocation of resources^6,7^. The high rates of readmission among patients with SCD was reported by Brousseau *et al*.^8^. The 30-day readmission rate was estimated to be 33.4%, with SCC as the cause of admission in 87% patients. Patients with public insurance were more vulnerable to readmission^8^. Other studies have also reported high rates of readmission and have highlighted several associated factors^9–14^. Yet, several questions remain unanswered. First, it is not clear if readmission rates are higher in the first week after discharge and merit specific attention to reduce the overall risk. Secondly, do the principal diagnoses underlying readmissions during the first week vary significantly from later readmissions? Third, what is the economic burden resulting from readmission in the United States? And finally, what are the modifiable patient or hospital-related factors that independently predict the risk of readmission and could be targets for the future interventions?

The aims of this study are^1^ to estimate the unplanned all-cause 30-day and 7-day readmission rates among patients admitted with SCC^2^, to identify the risk factors independently associated with readmissions, and^3^ to assess the impact of readmission on patient outcomes and resulting financial burden on the health care system.

## Methods

For this retrospective cohort study, we analyzed data from Agency for Healthcare Research and Quality’s Healthcare Cost and Utilization Project (HCUP) Nationwide Readmission Database (NRD) for the year 2016^15^. This study utilized deidentified data collected and distributed by HCUP through a third-party vendor and therefore does not require an institutional board review (IRB) approval or consent from the individual patients. The description of the database, study population, variables and statistical methods are provided as supplementary material. We applied STATA, version 15.0 (StataCorp, College Station, TX) for the statistical analysis in this study. We plotted probability of readmission against time from discharge to readmission by applying Kaplan Meier (KM) survival curve. Patients were censored at day 30 if they had not experienced the event of interest, all-cause readmission.

### Study outcomes

The primary outcome of this study was 30-day all-cause readmission. In NRD, each patient is assigned a unique identification number which facilitates identification of all index and readmissions within the state. The primary outcome was defined as any admission for a non-traumatic cause within 30 days of the index admission. We included only the first readmission for patients with multiple readmissions within 30 days of discharge. For calculation of readmission rates, patients who were discharged alive after the index admission (after excluding patients who died during the index admission) were used as denominator. The secondary outcomes were (a) 7-day all-cause hospital readmission (b) the ten most common principal diagnoses for 30-day and 7-day readmission (c) healthcare resource use attributed to readmission: length of stay (LOS), total hospitalization costs and charges (d) predictors for 30-day and 7-day readmission (e) in-hospital mortality during index admissions (f) 30-day mortality rate for index admissions (f) in-hospital mortality during readmission

### Declaration

This study involving human participants was conducted in accordance with the ethical standards of the institutional and/or national research committee and with the 1964 Helsinki declaration and its later amendments or comparable ethical standards.

## Results

### Patient characteristics

**Figure 1:**
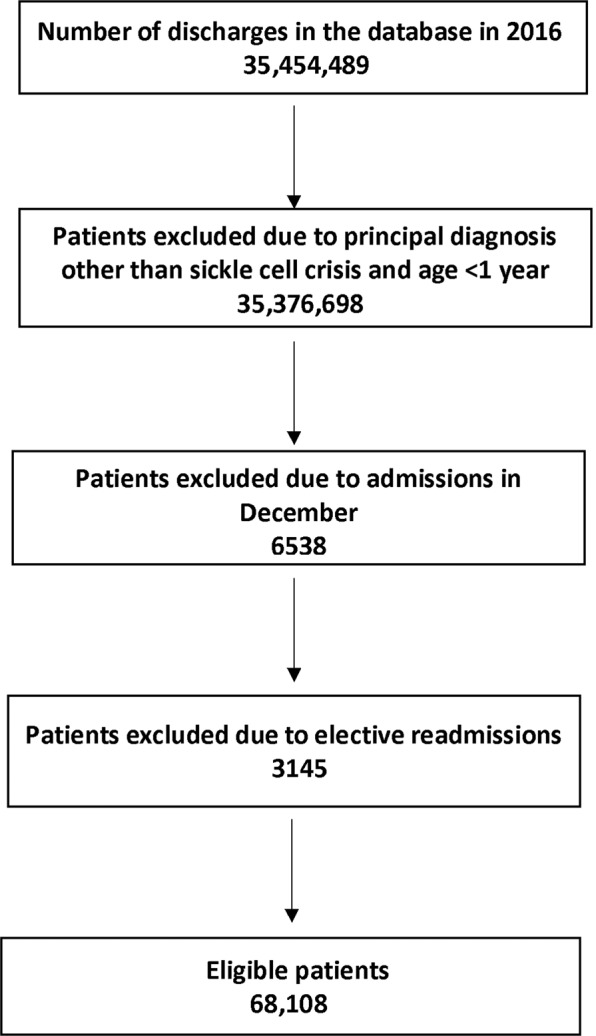
Flow diagram showing selection of the eligible patients.

Figure 1 shows the flow diagram for inclusion of eligible patients.

Of the 35,454,489 hospitalizations in the US, 68,108 index admissions for SCC were eligible for the study. Table 1 shows the characteristics of the patients and the treating facilities. The mean age of patients was 27.5 (CI:26.2–28.8) years, and 53.9% of patients were female. Medicaid was the most common insurance in 53.4%. Approximately 90% of the patients had a Charlson comorbidity index (CCI) of 0 or 1. Almost one-half of the patients belonged to the lowest income quartile and over two-thirds lived in large metropolitan areas with at least 1 million residents. Most patients were treated at large, urban and teaching hospitals. A comparable proportion was also treated at hospitals with the highest volume quintile.

**Table 1:** Characteristics of study patients.

Variables	N = 68,108 (100)
**Gender**	
Female	36,710 (53.9)
Male	31, 398 (46.1)
**Mean age in years**	27.5
**Primary Payor (Insurance)**	
Medicaid	17,568 (25.79)
Medicare	36,389 (53.43)
Private	12,040 (17.68)
Uninsured	2111 (3.10)
**Charlson comorbidity index**	
0	43,405 (63.73)
1	18,229 (26.77)
2	3,762 (5.52)
≥ 3	2,712 (3.98)
**Median Income in the zip code**	
<$43,000	35,384 (51.95)
$43,000-$51,999	15,758 (23.14)
$54,000-$70,999	11,075 (16.26)
≥ $71,000	5,891 (8.65)
**Type of residence**	
Large metropolitan (≥1 million residents)	47,546 (69.81)
Small metropolitan (<1 million residents)	17,391 (25.53)
Micropolitan	2,243 (3.29)
Non-urban	928 (1.36)
**Hospital size**	
Small	8,045 (11.81)
Medium	16,980 (24.93)
Large	43,083 (63.26)
**Hospital volume quintiles**	
1.(Lowest)	554 (0.81)
2.	1,659 (2.44)
3.	4,079 (5.99)
4.	9,930 (14.58)
5.	(Highest) 51,886 (76.18)
**Hospital Location**	
Rural	11,588 (17.01)
Urban	56,520 (82.99)
**Discharge against medical advice**	1944 (2.86)
**Hospital teaching status**	
Non-teaching	14,759 (21.67)
Teaching	53,349 (78.33)

### Thirty-Day all-cause hospital readmission

**Figure 2:**
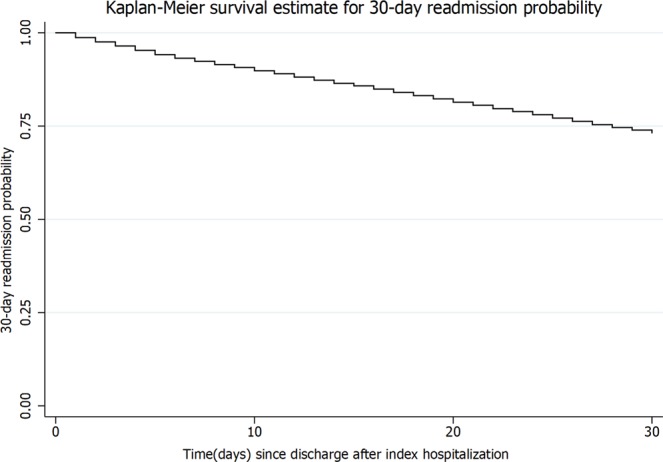
Kaplan Meier plot showing the probability of 30-day readmission after discharge.

Of 67,887 (99.7%) patients who were discharged alive after the index hospitalization, 18,099 were readmitted within 30 days, leading to a 30-day all-cause readmission rate of 26.9%. In the Fig. 2, the KM curve shows the probability of 30-day readmission. Overall, the total time at risk was 5,520,433 days. The first readmission occurred on day 0 while last admission occurred on day 30. Of the 10 most common principal diagnosis leading to readmission within 30 days, 7 were related to SCC which contributed to 86% readmissions (Table 2).

**Table 2:** Top ten principal diagnosis leading to 30-day readmission.

Principal diagnosis	ICD-10 code	N (%)
Hb-SS disease with crisis, unspecified	D5700	12,597 (69.6)
Hb-SS disease with acute chest syndrome	D5701	959(5.3)
Sickle-cell/Hb-C disease with crisis, unspecified	D57219	670(3.7)
Sickle-cell thalassemia with crisis, unspecified	D57419	615(3.4)
Other sickle-cell disorders with crisis, unspecified	D57819	253(1.4)
Sepsis, unspecified organism	A419	253(1.4)
Sickle-cell thalassemia with acute chest syndrome	D57411	253(1.4)
Sickle-cell disease without crisis	D571	217(1.2)
Pneumonia, unspecified organism	J189	72(0.4)
Other pulmonary embolism without acute cor pulmonale	I2699	72(0.4)

### In-hospital and 30-day mortality rates

Of the 68,108 index admissions, 211 patients died during the index hospitalization while an additional 44 patients died during the next 30 days, leading to an in-hospital and 30-day mortality rate of 0.31% and 0.37%, respectively.

### In-hospital mortality rate among readmitted patients

Among 18,099 patients who required readmission within 30 days, 70 (0.39%) patients died during the second hospitalization which was not significantly different from the mortality during index hospitalizations, *p* = *0.11*.

### Morbidity among readmitted patients

Significantly higher proportion of patients developed shock during rehospitalization compared to the index hospitalization, but there was no difference in the proportion of patients requiring mechanical ventilation and blood transfusions (Table 3).

**Table 3:** Comparison of morbidity and resources utilization during index hospitalization and 30-day readmission.

Morbidity	Index Hospitalization	30-day Readmission	*p* value
Shock n (%)	95(0.14)	69(0.38)	**<0.01**
Mechanical Ventilation n (%)	347(0.51)	107(0.59)	0.17
RBC Transfusion n (%)	1696(2.49)	465(2.57)	0.65
**Resources utilization**			
LOS in days mean (95%CI)	4.9 (4.8–5.1)	5.3 (5.1–5.5)	**<0.01**
Total cost mean (95%CI)	$8064 (7679–8448).	$8485 (8072–8898)	**<0.01**
Total Charge mean (95%CI)	$31,625 (29,656–33,595)	$33,923 (31,955–35,890)	**<0.01**

### Resource use incurred from readmission

The mean LOS, total hospitalization costs and total hospitalization charges were significantly higher for the 30-day readmission cohort compared to index hospitalization (Table 3). The cumulative total length of hospitalization for all patients readmitted within 30 days of discharge was 95,445 days, which resulted in a total hospitalization costs of $152 million and total hospitalization charges of $609 million.

### Independent predictors of 30-day readmission

**Figure 3:**
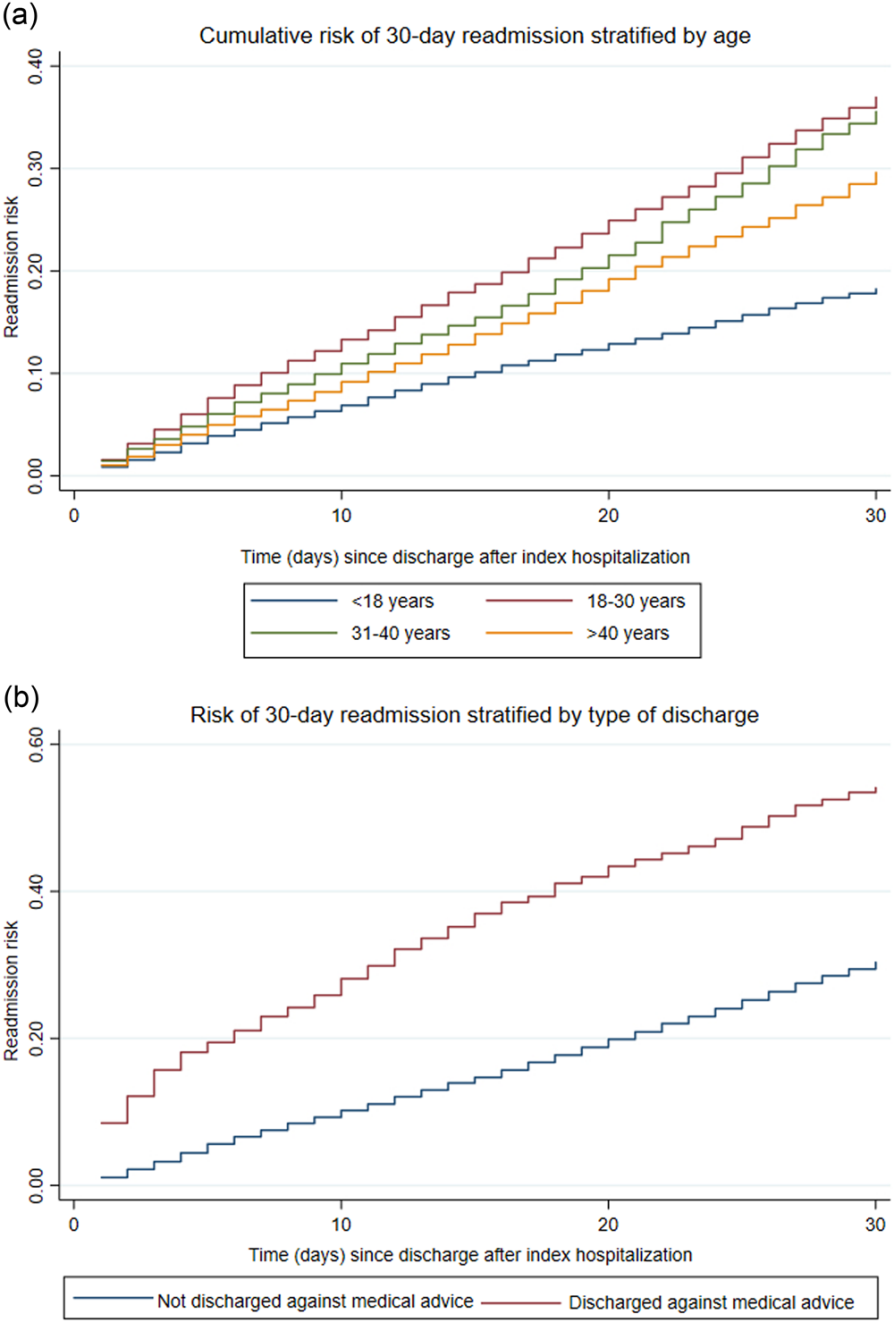
Risk of 30-day readmission by (**a**) age categories (**b**) discharge type.

In Fig. 2, the Kaplan-Meier curve shows the probability of 30-day readmission. A univariate cox regression analysis was applied to analyze the association of readmission with multiple variables (supplementary). The variables which were significantly associated with readmissions on univariate analysis were fitted into cox multivariate analysis to assess the strength of association independent of other variables. The final model is presented in Table 4. The variables which were found to be independent predictors of 30-day readmission were age, discharge against medical advice (AMA), higher CCI and the highest volume hospitals. Figure 3a,b show the risk of readmission by age categories and discharge types. The variables that were associated with lower odds of 30-day readmission were the private insurance or being uninsured, zip code with higher income and use of mechanical ventilation. No other variables had influence on 30-day readmission.

**Table 4:** Factors affecting 30-day readmission on cox proportional hazard multivariate analysis.

Variables	Adjusted Odds Ratio	95% CI	*p* value
Age (continuous)	1.01	1.00–1.01	**<0.001**
Age (categorical)			
<18 years		Reference	
18–30 years	2.02	1.81–2.25	**<0.001**
31–40 years	1.90	1.70–2.11	**<0.001**
>40 years	1.54	1.36–1.74	**<0.001**
**Insurance type**			
Medicare		Reference	
Medicaid	1.02	0.96–1.09	0.54
Private	0.79	0.72–0.87	**<0.001**
Uninsured	0.65	0.54–0.79	**<0.001**
**Charlson comorbidity score**			
0		Reference	
1	1.16	1.1–1.23	**<0.001**
2	1.26	1.14–1.40	**<0.001**
≥3	1.42	1.25–1.60	**<0.001**
**Median income in the patient’s zip code**			
≤$38,999		Reference	
$39,000-$47,999	0.97	0.89–1.05	0.39
$48,000–$62,999	0.89	0.81–0.97	**0.01**
≥$63000	0.84	0.75–0.94	**<0.001**
**Hospital location**			
Rural		Reference	
Urban	0.88	0.76–1.01	0.08
**Ventilatory support**			
No		Reference	
Yes	0.44	0.26–0.74	**<0.001**
Length of stay (continuous)	1.01	1.00–1.01	0.21
**Teaching hospital**			
No		Reference	
Yes	1.04	0.91–1.19	0.59
**Hospital volume quintiles**			
1(lowest)		Reference	
2	1.30	0.94–1.79	0.11
3	1.50	1.12–2.02	**0.01**
4	1.57	1.18–2.10	**<0.01**
5 (highest)	1.66	1.25–2.21	**<0.01**
Discharge against medical advice	1.72	1.50–1.97	**<0.001**

### 7-day readmission risk and resulting health care utilization among patients with SCC

A subgroup analysis was conducted to study the underlying factors correlated with 7-day readmission risk. In this cohort, 5166 (7.6%) patients were readmitted within 7 days of discharge. The 10 principal causes for readmission are listed in Table S2. SCC and related diagnoses contributed to 4,323 (83.7%) readmissions. On multivariate analysis, the highest odds of readmission were observed in patients who developed shock (OR 2.96), followed by those who got discharged AMA (OR 2.89). As compared to pediatric patients, higher odds were observed for age groups 18–30 and 31–40 years. Female gender was associated with lower odds of 7-day readmission. Table (S3).

### 30-day readmission risk and resulting health care utilization among pediatric patients with SCCN

We analyzed the readmission rates and resulting excess health care utilization by pediatric patients with a principal diagnosis of SCC. A total of 15,740 weighted discharges were eligible. The mean age was 10.6 years. The primary insurance was Medicaid in 77.9%. We did not estimate CCI or AMA among pediatric patients. The characteristics of the study population is shown in Table S4. Of 15,732 patients discharged after the index hospitalization, 2,629 (16.7%) patients were readmitted within 30 days. The most common principal diagnoses for readmission were SCC or related diagnoses in 2170 (74.1%) patients (Table S5). The mean total charge ($29,593 vs $25,727, *p* < 0.001), mean total cost ($8070 vs $7284, *p* = 0.02) and mean LOS (4.3 (4.1–4.6) vs 3.9 (3.7–4.1) days, *p* < 0.001) for the readmitted patients were significantly higher than for the index admissions. The readmission of pediatric patients added an extra 11,368 days of hospitalization, resulting in a total cost of $21.1 million and a total charge of $77.3 million. On multivariate analysis increasing age (OR 1.06), developmental delay (OR 1.48) and bronchial asthma (OR 1.19) were associated with increased likelihood of 30-day readmission. (Table S6)

## Discussion

The present study using the nationwide readmission database demonstrated a 30-day-all cause readmission rate of 26.9% among patients admitted with a principal diagnosis of SCC in the US. The principal cause of readmission among 86% of these patients was SCC or a related diagnosis. The 30-day readmissions incurred 95,445 extra days of hospitalization in 2016, $152 million in total hospitalization costs, and $609 million in total hospitalization charges. The readmission rates were evenly distributed over 30-day period post discharge and the pattern of diagnosis leading to readmission within the first week were not different than the overall 30-day period.

To our knowledge, this is the first large-scale study in recent times conducted on a database that is an accurate representation of the national estimates of readmission. A previous study conducted on patients with SCC from eight state inpatient databases (SID) in 2005–2006 concluded a 30-day readmission rate of 33.2%, which is slightly higher than this study^8^. Although it’s likely that readmission rates among patients with SCC are declining, the differences in databases and study criteria make a comparison difficult. The features of NRD afford us a more accurate estimate of readmissions than previous databases^15,16^. Although the NRD data comes from individual SIDs, NRD is specifically designed to calculate readmission rates. The NRD 2016 consists of data from 27 states which are geographically uniformly distributed, ensuring that estimates are nationally representative^15^. We used ICD-10 codes which are more precise than ICD 9 codes used in the previous studies^17,18^. Also, we included only unplanned readmissions as only they are quality metrics of interest^19^. However, we could not capture patients who were readmitted in different states, leading to more conservative estimates. Several single center studies report 30-day readmission rates of 30%–50%^9,10^.

The readmission rate of 26.9% in our study is higher than reported in other illnesses like gastrointestinal bleeding, pneumonia and congestive heart failure, and resulted in significant increases in healthcare resource utilization^20–22^. 30-day readmission added an extra 95,445 days of hospitalization. Preventing these readmissions could have saved $152 million in hospital costs, $609 million in charges. Most was borne by public insurance, proof that maintaining the quality of healthcare delivery while keeping costs low is a challenge. A prior study on Florida Medicaid patients with SCC estimated a total lifetime expenditure of approximately $1 billion^23^. However, it did not specify total charges and costs^24^. There are no estimates of the contribution of readmission towards total cost, prior to our study.

In the present study, we focused on readmission risk at 30 days but also estimated 7-day readmission risk to identify a potential period of further vulnerability. Variations in readmission rates within the 30-day period have been reported previously for pneumonia and CHF^20^. Patients with pneumonia are at higher risk of readmission soon after discharge while CHF patients are at elevated risk towards the end of the 30 days. However, we did not observe such a variation with SCC where risk was evenly distributed over 30-day period. This may explain the marginal benefit of interventions targeted to inpatients like verbal discussion to improve patients’ understanding and compliance with an action plan, individualized pain plans in emergency department and discharge calls at 24 and 72 hours^25,26^. Based upon our findings, more effective measures warrant both inpatient and outpatient interventions beyond the initial follow-up visit. Ambulatory providers should be wary of the heightened risk of readmission throughout the first month post discharge.

The overall pattern in principal diagnoses leading to readmission within the first 7 days was not different from those over a 30-day period. The most common diagnoses leading to readmissions were related to SCC in >80% patients both at 7 and 30 days. This is consistent with previously reported factors leading to readmissions including premature discharge with ongoing crisis, prescribing inadequate pain medications, and rapid tapering of pain medications just prior to discharge^9,27,28^. Providers need to examine alternate strategies such as designing interventions based upon pain scale measurements, involving pain management teams, and close follow-up of patients in SCC and pain specialty clinics.

In this study, several potentially modifiable patient- and hospital-specific characteristics were identified as independently associated with readmission. While 30-day readmission rate is used by CMS as a quality metric, 7-days readmission risk has been suggested a better marker of inpatient care^29^. In this study, hospital related factors were related to 30-day readmission risk but none with 7-day readmission risk. Factors consistently associated with higher risk of readmission at 7 days and 30 days were age and AMA discharges.

The most vulnerable age group was 18–30 years, a finding also reported in a previous study^8^. This group is considered at higher risk because outpatient care is not as well organized as compared to the pediatric age group. The care they receive is generally less comprehensive than that to which they were formerly accustomed. During this period of transition in care, sickle cell patients have higher acute care usage, hospitalization and readmission rates^30,31^. While leaving hospital AMA has been reported as a risk factor for readmission in several other conditions, including HIV and bronchial asthma it has not been studied on a large scale among patients with SCC^32^. Some 2.4% of patients left AMA and were 2.9 and 1.8 times more at risk of readmission at 7-day and 30-day respectively. Two previous studies analyzed factors underlying self-discharge AMA. In the first, 46.5% of subjects reported leaving AMA. Of those, 65% patients also reported difficulty in persuading their healthcare providers (HCP) about their pain and need for more pain medication^33,34^. The management of pain is challenging due to lack of objective criteria^35,36^. Patient mistrust, patient-HCP conflict, and suboptimal communication between physician and patient have been identified as potential contributors^33,37–41^. Poor quality interactions between patients and HCPs has been reported as the most consistent predictor of future mistrust. Studies have highlighted high rates of patients reporting disbelief about their pain levels^41^. HCPs should engage patients with care planning, pain specialists and hematologists to mitigate self-discharge rates. Self-discharge on patients’ record should alert physicians in a point-of-service manner.

Other factors which were associated with higher risk of 30-day readmission included higher CCI, low socioeconomic status (SES) and patients treated at high volume centers. The CCI is a reflection of the global burden of comorbidity. The higher risks among SCC patients with higher CCI have been reported previously^42^. For some patients, higher readmission rates are a direct consequence of their comorbidities and increased frailty^43^. We used the CCI for adult patients but selected common comorbidities for pediatric patients. Besides age, asthma and developmental delays were the only predictors of 30-day readmission among the pediatric age group a finding similar to previous studies^44,45^. Likewise, higher risk among low SES has been reported, which could be related to less access to healthcare or more severe disease^42^. The higher rate of readmission at high volume centers may reflect the fact that tertiary and larger secondary hospitals have disproportionately greater in-patient volume, and more complicated patients^46^. It may be that a more complicated admission is likely to be followed by a readmission, as high volume may hinder high quality transitional care. Lower mortality rates offer more opportunity for readmission and vice-versa which could explain the lower readmission rates seen among small number of mechanically ventilated patients in this study. Moreover, it is possible that due to longer LOS, the pain crisis was already resolved before discharge.

Another interesting finding in this study was the lower risk of readmission among uninsured patients as compared to patients with Medicare/Medicaid. This lower risk of readmissions among uninsured individuals has also been noted in other illnesses^22^ and is not fully understood since patients who are severely ill are eligible for emergency Medicaid programs^47^.

The findings from this study should be interpreted in the light of following limitations. NRD is an administrative database with no information on disease severity, outpatient follow-up or opioid prescriptions. Database studies are also susceptible to missing or erroneous codes^48^. The NRD only has data for one calendar year and does not capture readmission data on readmissions in a different state. However, this study includes a very large sample size and utilized ICD-10, allowing us to include only those patients who were diagnosed with SCC on discharge. There is no information on the severity of sickle cell crisis or on therapeutic regimens in NRD therefore their impact on readmission could not be analyzed in this study. Also, the impact of specialized SCC clinics on readmission rates could not be studied due to lack of this data in NRD. However, we could still categorize the treatment facilities based upon their location (rural-urban) and by volume of SCC patients treated at each facility (into quintiles). NRD is specifically designed to study readmission rates and is nationally representative of all US hospitals. Unique variable selections such as hospital costs, household income, and hospital characteristics are not available in single institution studies.

In conclusion, we used NRD to estimate unplanned 30-day all-cause readmission rates among patients with SCC, patient and hospital factors influencing readmissions, the resulting health care utilization, and its impact on patient outcome. We determined that the 30-day readmission rate is 26%. This contributed a significant burden to healthcare resources: an additional 95,445 days of hospitalization, resulting in $152 million cost of hospitalization and $609 million in total hospitalization charges in 2016. The current study highlights several patient- and hospital-related characteristics which could be targeted to reduce the readmissions: age, leaving hospital AMA, comorbidities, hospital volume and SES. Although not all factors are amenable to intervention, they can identify patients who are at higher risk of rehospitalization for closer surveillance. Future studies on intervention to reduce readmission should target high-risk patients.

## Supplementary information

Supplementary Information.

## Data Availability

The data that support the findings of this study are available from. The Nationwide Readmissions Database (NRD) which is a set of inpatient databases in the health care utilization project (HCUP) family designed for readmission analyses. These HCUP databases are created by AHRQ through a Federal-State-Industry partnership but restrictions apply to the availability of these data, which were used under license for the current study, and so are not publicly available. Data are however available from the authors upon reasonable request and with permission with permission of HCUP.
