# Supplementary material for: Epidemiology and Predictors of all-cause 30-Day readmission in patients with sickle cell crisis
Source: Sci Rep. 2020 Feb 7;10:2082. doi: 10.1038/s41598-020-58934-3 (PMC7005718; doi:10.1038/s41598-020-58934-3)
Supplement: Supplementary file 1 — Supplementary Information. [file 41598_2020_58934_MOESM1_ESM.pdf]

METHODS

Database

For this retrospective cohort study, we analyzed the data from Agency for Healthcare Research and Quality's (NHRQ) Healthcare Cost and Utilization Project (HCUP) Nationwide Readmission Database (NRD) for the year 2016(15). This is the largest all payer readmission database in the United States which is publicly available. In 2016, the NRD contained data from 27 geographically dispersed HCUP partner states, accounting for 56.6% of all United States hospitalizations and 57.8% of the total United States population(15). Unweighted, NRD has data on roughly 17.2 million discharges which estimates to 36 million on applying discharge weights.

The data for NRD is collected by applying stratified probability sampling method and therefore it is representative of all nonfederal acute care inpatient hospitalizations in the United States. In summary, hospitals which are contributing data are stratified based upon ownership, number of beds, geographic region, urban/rural location, and teaching status. From each stratum, a 20% probability sample of all hospitals is collected. Finally, discharges from these hospitals are recorded with data on individual patients including demographics, principal and secondary diagnoses, vital status at discharge, readmission, cause of readmission and data on health care resources utilization including length of stay during index hospitalization and readmission, interventions performed (mechanical ventilation and blood transfusion) and total hospitalization costs and charges are recorded. To make it nationally representative, a certain weight is applied to each discharge which is estimated by dividing the total number of discharges from all acute care hospitals in the United States by the number of discharges included in the 20% sample. The NRD has data on both patient and hospital characteristics. In 2016 NRD, up to 25 discharge diagnoses

and 15 procedures are recorded for each discharge using the International Classification of Diseases, tenth Revision, Clinical Modification (ICD10-CM) (15, 16).

Study Population

Although, sickle cell pain episode is a preferable term to sickle cell crisis these days we used pain crisis due to the ICD-10 coding system which has multiple diagnosis codes for sickle cell crisis. For this study, we included patients who were discharged with a principal diagnosis of sickle cell crisis(16). Patients were excluded if they were younger than 1 year or if the admission was elective. This age cut-off was used to make it comparable to the previous study which was conducted on using states databases(8). Patients in the NRD are captured in each calendar year from January 1st to December 31st without any linkage to the previous or following years. Therefore, we did not include patients with index hospitalization discharges happening in the month of December similar to other studies conducted on using this database(17). This study was based upon a publicly available deidentified data and is therefore exempted from the institutional review board approval.

Description of Variables

We extracted data on age (in years), gender, median household income for patient's zip code ($1–$42,999, $43,000–$53,999, $54,000–$70,999, and $71,000 or more), primary payer (Medicare, Medicaid, private insurance, and uninsured), patient residence (large metropolitan areas with at least 1 million residents, small metropolitan areas with less than 1 million residents, micropolitan areas (nonurban residual), and not metropolitan or micropolitan), discharge to a rehabilitation facility, hospital size based on number of beds (small, medium, and large), and teaching status. The principal diagnosis, patient comorbidities, procedures performed during hospitalization (blood transfusion), and mechanical ventilation were identified using the appropriate ICD10-CM codes. To assess the comorbidity burden, Charlson-Deyo comorbidity scores were calculated and based

upon these scores, patients were stratified into 4 groups (0, 1, 2 3+)(18). To estimate the hospital volume, we added all the weighted admissions with sickle cell crisis admitted at each hospital during 2016 and divided them into quintiles (five equal groups) for this study. The 10 most common reasons for readmission were determined by the principal diagnosis for readmission on each patient. The length of stay was directly calculated from the NRD and analyzed as a continuous variable. Likewise, total hospital charges and total costs are directly recorded in the NRD which were also analyzed as continuous variable. The total hospital charge is the amount billed by the hospitals for the entire hospitalization, but it may not reflect the actual cost of health care. On the other hand, the total hospitalization cost reflects the true cost of health care. To calculate the total cost, we multiplied the total hospital charges by the corresponding hospital specific cost to charge ratio. The cost to charge ratio in the NRD is based upon all-payer inpatient cost and is provided to Centers for Medicare and Medicaid Services by all the hospitals (19). The in-hospital mortality rate during index admission and readmission was calculated using the patient vital status at discharge. The 30-day mortality rate was calculated by recording the vital status at discharge after any readmission within 30 days of discharge of index hospitalization. This approach did not capture any deaths at home or during transportation from hospital.

Statistical Analysis

We implemented weighting of patient-level observations to obtain the estimates which are nationally representative of the entire population of United States, hospitalized with sickle cell crisis. Continuous variables were compared using the student t test while Fisher exact test was used to compare the proportions. All *P* values were 2 sided, with 0.05 as threshold for statistical significance. Patients were censored at day 30, if they did not experience the event of interest which was all-cause readmission in this study. Univariable Cox regression analysis was used to

calculate unadjusted odds ratios for the primary outcome. To determine the risk factors which were independently associated with readmission, multivariable regression models were built by including all variables that were significantly associated with the readmission on univariate analysis. The variables which were analyzed for any association with readmissions included: 1, patient related: age, gender, insurance type, median income in patient's zip code, location of the patient's residence, and Charlson comorbidity score; 2, hospital related: hospital number of beds, teaching status, location, and sickle cell treatment volume (as quintiles); 3, treatment related: RBC transfusion; and 4, severity related: use of mechanical ventilation, shock, and length of stay. To account for the missing variables, we applied the multivariate imputation by chained equations (i.e., MICE) method which was estimated from sequential multivariable models with fully conditional specifications(20). We applied regression analysis on missing covariates and constructed imputed datasets using information from all the covariates with missing information as well as those covariates without missing data. Results after imputation were not significantly different than the original analysis and therefore, we reported results on analysis without imputation.

References

15. THE HCUP NATIONWIDE READMISSIONS DATABASE (NRD), 2010-2016 2019 [Available from: https://www.hcup-us.ahrq.gov/db/nation/nrd/Introduction_NRD_2010-2016.jsp.

16. Free 2019 ICD-10-CM Codes - ICD-10 Data https://www.icd10data.com/ICD10CM/Codes - Google Search2019. Available from: https://www.google.com/search?q=Free+2019+ICD-10-CM+Codes+-+ICD-10+Data+https%3A%2F%2Fwww.icd10data.com%2FICD10CM%2FCodes&oq=Free+2019+ICD-10-CM+Codes+-+ICD-10+Data+https%3A%2F%2Fwww.icd10data.com%2FICD10CM%2FCodes&aqs=chrome..69i57.2647j0j4&sourceid=chrome&ie=UTF-8.

17. Abougergi MS, Peluso H, Saltzman JR. Thirty-Day Readmission Among Patients With Non-Variceal Upper Gastrointestinal Hemorrhage and Effects on Outcomes. Gastroenterology. 2018;155(1):38-46 e1.
18. Deyo RA, Cherkin DC, Ciol MA. Adapting a clinical comorbidity index for use with ICD-9-CM administrative databases. Journal of clinical epidemiology. 1992;45(6):613-9.
19. HCUP-US Cost-to-Charge Ratio Files 2019 [Available from: https://www.hcup-us.ahrq.gov/db/state/costtocharge.jsp.
20. White IR, Royston P, Wood AM. Multiple imputation using chained equations: issues and guidance for practice. Statistics in medicine. 2011;30(4):377-99.

Table S1 List of ICD-10 codes used to identify patients with Sickle Cell crisis

D5700	D57412	D57419
D57811	D57812	D57819
D5701	D5702	D57211
D57411	D57212	D57219

Table S2 Top ten principal diagnosis leading to 7-day readmission

Principal diagnosis	ICD-10 code	N (%)
Hb-SS disease with crisis, unspecified	D5700	3382 (65.47)
Hb-SS disease with acute chest syndrome	D5701	359(6.95)

Sickle-cell/Hb-C disease with crisis, unspecified	D57219	179(3.47)
Sickle-cell thalassemia with crisis, unspecified	D57419	170(3.3)
Pneumonia, unspecified organism	J189	103(2)
Other sickle-cell disorders with crisis, unspecified	D57819	76(1.47)
Sickle-cell disease without crisis	D571	67(1.31)
Sepsis, unspecified organism	A419	61(1.18)
Sickle-cell/Hb-C disease with acute chest syndrome	D57211	38(0.73)
Acute kidney failure, unspecified	N179	29(0.55)

Table S3. Factors affecting 7-day readmission on cox proportional hazard multivariate analysis

**Variables**	**Odds Ratio**	**95% CI**	***p* value**
**Age (continuous)**	1.01	1.00-1.01	**<0.001**
**Age (categorical)**			
<18 years		Reference	
18-30 years	1.74	1.45-2.07	**<0.001**
31-40 years	1.31	1.06-1.61	**0.01**
>40 years	1.12	0.91-1.39	**0.3**
**Charlson comorbidity score**			
0		Reference	
1	1.2	0.9-1.36	0.41
2	1.08	0.87-1.37	.46
≥ 3	1.10	0.83-1.47	0.50
**Shock**	2.96	1.09-8.04	**0.03**
**Female Gender**	0.86	0.76-0.96	**0.01**
**Discharge against medical advice**	2.89	2.24-3.23	**<0.001**

Table S4. Characteristics of Pediatric patients

Variables	N=15,740 (100)
**Gender**	
Female	7,886 (50.1)
Male	7,854 (49.9)
**Mean age in years**	11.6 (11.3-11.9)
**Primary Payor (Insurance)**	
Medicare	12 (0.08)
Medicaid	12,264 (77.9)
Private	3,275 (20.5)
Uninsured	189 (1.2)
**Median Income in the zip code**	
< $39,000	7,692 (48.9)
$39000-$47,999	4,004(25.4)
$48,000-$62,999	2,704 (16.9)
≥ $63000	1,341 (8.5)
**Type of residence**	
Large metropolitan (≥1 million residents)	11,306 (71.8)
Small metropolitan (<1 million residents)	4,127 (26.2)
Micropolitan	214 (1.4)
Non-urban	94 (0.6)
**Day of index admission**	
Weekday	12,912 (75.1)
Weekend	4,285 (24.9)
**Hospital size**	
Small	1,731 (12)
Medium	3,528 (22.4)
Large	10,481 (66.6)
**Hospital volume quintiles**	
1.(Lowest)	147 (0.9)
2.	321 (2)
3.	795 (5.1)
4.	2,057 (13.1)
5. (Highest)	12,419 (78.9)
**Hospital Location**	
Rural	1,057 (6.7)
Urban	14,683 (93.3)
**Hospital teaching status**	
Non-teaching	1,364 (8.7)
Teaching	14,376 (91.3)

**Comorbidities**	
Bronchial Asthma	4897(31.1)
Developmental Delay	765(4.9)
Obesity	341(2.2)
Epilepsy	162(1.03)

Table S5 Top ten principal diagnosis leading to 30-day readmission among pediatric patients

Principal diagnosis	ICD-10 code	N (%)
Hb-SS disease with crisis, unspecified	D5700	1385(44.6)
Hb-SS disease with acute chest syndrome	D5701	230 (7.4)
Sickle-cell/Hb-C disease with crisis, unspecified	D57219	207(6.7)
Sickle-cell thalassemia with crisis, unspecified	D57419	146(4.7)
Sickle-cell disease without crisis	D571	86(2.8)
Hb-SS disease with splenic sequestration	D5702	37(1.2)
Sickle-cell/Hb-C disease with acute chest syndrome	D57211	31(1)
Pneumonia, unspecified organism	J189	28(0.9)
Other sickle-cell disorders with crisis, unspecified	D57819	25(0.8)
Sickle-cell thalassemia with acute chest syndrome	D57411	23(0.7)

Table S6 Predictors of 30-day readmission among pediatric patients.

Variables	Hazard ratio	95% Conf. Interval	p value
Age	1.06	1.04-1.07	<0.001
Mechanical ventilation	0.22	0.03-1.83	0.16
Hospital Volume Quantiles			
1 (lowest)		Reference	
2	2.00	0.75-5.30	0.17
3	2.13	0.89-5.11	0.09
4	2.15	0.91-5.11	0.08
5 (highest	1.96	0.84-4.59	0.12
Developmental delay	1.48	1.10-1.99	0.01
Epilepsy	0.48	0.24-0.1.1	0.08
Bronchial Asthma	1.19	1.02-1.40	0.03
Large metropolitan (1 million residents)		Reference	
Small metropolitan (<1 million residents)	0.94	0.78-1.14	0.56

Micropolitan	0.55	0.28-1.07	0.08
Non-urban	0.76	0.19-2.99	0.70
